# Stable Gastric Pentadecapeptide BPC 157 and Intestinal Anastomoses Therapy in Rats—A Review

**DOI:** 10.3390/ph17081081

**Published:** 2024-08-17

**Authors:** Salem Bajramagic, Marko Sever, Fran Rasic, Mario Staresinic, Anita Skrtic, Lidija Beketic Oreskovic, Ivana Oreskovic, Sanja Strbe, Svjetlana Loga Zec, Josip Hrabar, Luka Coric, Matea Prenc, Vladimir Blagaic, Klara Brcic, Alenka Boban Blagaic, Sven Seiwerth, Predrag Sikiric

**Affiliations:** 1 Department of Pharmacology, School of Medicine, University of Zagreb, 10000 Zagreb, Croatia; salem.bajramagic@gmail.com (S.B.); dr.sever.marko@gmail.com (M.S.); fran.rasic@icloud.com (F.R.); ravnateljstvo@kb-merkur.hr (M.S.); lidijabeketicoreskovic@gmail.com (L.B.O.); psikiric@gmail.com (I.O.); strbes@gmail.com (S.S.); hrabri91@gmail.com (J.H.); luka.coric3105@gmail.com (L.C.); prenc.matea2@gmail.com (M.P.); vladimir.blagaic@gmail.com (V.B.); klara.brcic@kbcsm.hr (K.B.); abblagaic@mef.hr (A.B.B.); 2 Clinic of General and Abdominal Surgery, Clinical Center University of Sarajevo, 71000 Sarajevo, Bosnia and Herzegovina; 3 Department of Surgery, School of Medicine, University of Zagreb, 10000 Zagreb, Croatia; 4 Department of Pathology, School of Medicine, University of Zagreb, 10000 Zagreb, Croatia; sven.seiwerth@mef.hr; 5 Institute of Pharmacology, Clinical Pharmacology and Toxicology, Faculty of Medicine, University of Sarajevo, 71000 Sarajevo, Bosnia and Herzegovina; svjetlana.loga@gmail.com; 6 Department of Diagnostic and Interventional Radiology, University Hospital Centre, 10000 Zagreb, Croatia

**Keywords:** stable gastric pentadecapeptide BPC 157, esophagogastric, colocolonic, jejunoileal, and ileoileal anastomoses, short bowel, rats, therapy

## Abstract

By introducing the healing of many distinctive anastomoses by BPC 157 therapy, this review practically deals with the concept of the resection and reconnection of the hollow parts of the gastrointestinal tract as one of the cornerstones of visceral surgery. In principle, the healing of quite distinctive anastomoses itself speaks for applied BPC 157 therapy, in particular, as a way in which the therapy of anastomoses can be successfully approached and carried out. Some of the anastomoses implicated were esophagogastric, colocolonic, jejunoileal, and ileoileal anastomoses, along with concomitant disturbances, such as esophagitis, sphincter dysfunction, failed intestinal adaptation, colitis, short bowel syndrome, major vessel occlusion, NO-system, and prostaglandins-system dysfunction, which were accordingly counteracted as well, and, finally, findings concerning other anastomoses healing (i.e., nerve and vessel). Moreover, the healing of fistulas, both external and internal, colocutaneous, gastrocutaneous, esophagocutaneous, duodenocutaneous, vesicovaginal, colovesical, and rectovaginal in rats, perceived as anastomoses made between two different tissues which are normally not connected, may also be indicative. This may be a particular reconnection of the parts of the gastrointestinal tract to re-establish adequate integrity depending on the tissue involved, given that both various intestinal anastomoses and various fistulas (intestinal and skin were accordingly healed simultaneously as the fistulas disappeared) were all healed.

## 1. Introduction

This review aims to reveal the particular significance of the stable gastric pentadecapeptide BPC 157 for anastomosis healing (previously employed in ulcerative colitis, no reported toxicity, lethal dose (LD1) not achieved), particularly intestinal anastomosis [1,2,3,4,5,6,7,8,9,10,11]. Considering the important issue of intestinal anastomosis healing [12], the supportive evidence for BPC 157 therapy in anastomosis healing was already reviewed, focusing particularly on skin wound therapy, incisional/excisional wounds, deep burns, diabetic ulcers, and alkali burns, which may be generalized to other tissue healing [2]. 

Practical applicability (i.e., given alone, with the same dose range and same equipotent routes of application, regardless of the injury tested) is likely due to its original cytoprotection background [1,2,3,4,5,6,7,8,9,10,11]. Being native and stable in human gastric juice for more than 24 h (unlike standard growth factors rapidly destroyed within minutes, i.e., h-TGF, and h-EGF), it acts as a cytoprotection mediator, translating the maintenance of stomach and gastrointestinal tract epithelial and endothelial integrity to the healing of other tissues [1,2,3,4,5,6,7,8,9,10,11]. Thereby, there is a pleiotropic beneficial effect, combining the simultaneous healing of different tissues and particular wound healing capabilities [2]. Noteworthy is the point of the simultaneous healing of different tissues, a point specifically emphasized in the original introduction of the concept of cytoprotection [13,14] and later extended by the application of the cytoprotective agents to the organoprotection concept [15] [4,8]. The extent of such healing and conceptual significance illustrates the healing of the fistulas that would spontaneously hardly heal (colocutaneous, gastrocutaneous, esophagocutaneous, duodenocutaneous, vesicovaginal, colovesical, and rectovaginal) in rats and the potency of BPC 157 for both external and internal fistulas, as reviewed [3].

Consequently, stable gastric pentadecapeptide BPC 157 may have a particular background for anastomosis healing. Namely for adequate wound healing [2] and thereby, in particular, intestinal anastomosis [12], the important theoretical and practical point concerns the healing of the wounds as a whole. This would accomplish the resolution of vessel constriction, the primary platelet plug, the fibrin mesh, which acts to stabilize the platelet plug, and the resolution of the clot [2]. Indeed, as a confirmation of this general concept, BPC 157 is effective in wound healing [2] much like it is effective in counteracting bleeding disorders, produced by amputation, and/or anticoagulant and antiplatelet agent application [16,17,18]. Likewise, BPC 157 may prevent and/or attenuate or eliminate and, thus, counteract both arterial and venous thrombosis [19,20,21,22,23,24,25,26,27,28,29,30,31,32,33,34,35,36,37,38,39]. Together, these highlight therapy-proof endothelium maintenance/recovery [13,14] to the activation of collateral rescuing pathways as being key for the pleiotropic beneficial effect of cytoprotective agents that BPC 157 therapy might rapidly induce [19,20,21,22,23,24,25,26,27,28,29,30,31,32,33,34,35,36,37,38,39]. Then, confronted with obstructed vessels, there is a circumvention of the occlusion, “bypassing key”, upgrading minor vessels to take over the function of the disabled major vessel, activating collateral rescuing pathways (i.e., azygos vein direct blood flow delivery) to re-establish reorganized blood flow [19,20,21,22,23,24,25,26,27,28,29,30,31,32,33]. This may be the particular action of BPC 157 in ischemia/reperfusion. The demonstration was a counteraction of the occlusion/occlusion-like syndromes, induced by procedures that all severely affect endothelium function, resulting in vascular and multiorgan failure in severely affected rats. There are peripherally and centrally permanent major vessel occlusions, arteries, and veins [19,20,21,22,23]. Also, this occurs with similar harmful procedures [27,28,29,31] or distinctive agent applications [24,25,26,30,32,33]. Severe lesions in the brain (intracerebral, intraventricular hemorrhage) and heart (congestion, myocardial infarction, severe arrhythmias) and congestion/hemorrhage in the lungs, liver, kidney, and gastrointestinal tract consistently present as a whole these severe vascular and multiorgan failures. The inferior caval vein and the superior mesenteric vein were congested, and the azygos vein collapsed. Verifying the failed major vessels, widespread venous and arterial thrombosis, and stasis peripherally and centrally consistently advanced the widespread Virchow triad circumstances. Severe blood pressure disturbances (i.e., intracranial (superior sagittal sinus), portal, and caval hypertension and aortal hypotension) consistently occurred [19,20,21,22,23,24,25,26,27,28,29,30,31,32,33]. As the consequence of the activated collateral pathway (i.e., direct blood flow delivery via azygos vein) by BPC 157 therapy, all of these disturbances as occlusion/occlusion-like syndromes were attenuated/eliminated as a whole [19,20,21,22,23,24,25,26,27,28,29,30,31,32,33].

Essential support for such a particular vascular effect could be from the interaction with the systems essentially involved in cytoprotection and vessel functioning, and for the NO-system [8,17,34,35,36,37] and prostaglandins system [38], as a whole. The NO release [34,35] as a modulatory controlling effect substantiates the counteraction of the adverse effects of either NOS blockade (i.e., L-NAME-hypertension and the pro-thrombotic effect) or NOS over-stimulation (i.e., L-arginine-hypotension and the anti-thrombotic effect) [7,17,34]. These may be essential for a particular vascular recovery. Likewise, there were counteractions to the large range of adverse effects of NSAIDs, both non-specific and specific, and COX-1 and COX-2 inhibitors (i.e., ulceration, liver and brain injuries, and bleeding) [38]. Furthermore, thrombocyte function maintenance without affecting coagulation pathways [16,17,18], particularly controlling vasomotor tone through the activated Src-Caveolin-1-eNOS pathway [36,37], can ascertain that these counteractions occur as a particular effect. Finally, Fourier transform infrared spectroscopy directly demonstrated the instant effect of BPC 157 therapy in the vessel wall, the rapid change in the lipid contents, and the rapid change in protein secondary structure conformation [39]. This can be a conclusive indicator that BPC 157 rapidly ascertains vessel functioning even in the worst circumstances, which is an important point for anastomosis healing.

## 2. Anastomosis Healing

Together, this evidence (the references were the primary source of the prior research) would provide a review of a consistent background for BPC 157, particularly its effect on various anastomosis healings in different parts of the gastrointestinal tract and, thereby, its possible clinical significance. Thus, the evidence will show whether this background of BPC 157 would overwhelm the general limitation, such as the existing lack of knowledge about the basics of the process of anastomotic wound healing in the gastrointestinal tract, given that the concept of the resection and reconnection of the hollow parts of the tract is one of the cornerstones of visceral surgery [12]. Here, as a strength of evidence, in addition to resolving regular complications, such as anastomosis leak, dehiscence, and mentioned fistulas [12], BPC 157 therapy would provide additional evidence for esophagogastric [40], jejunoileal [41,42], ileoileal [43], and colocolonic [44,45] anastomoses healing as a whole. Notably, BPC 157 involves the counteraction of the other specific complications that would otherwise arise from the anastomosis malfunctioning and failing integrity of particular parts of the gastrointestinal tract depending on the site of anastomosis [40,41,42,43,44,45,46]. Furthermore, other anastomoses (i.e., sciatic nerve, abdominal aorta) were healed [47,48].

## 3. Anastomoses in the Upper Part of the Gastrointestinal Tract

### Esophagogastric Anastomosis

Esophagogastric anastomosis and esophagogastric anastomosis healing by BPC 157 therapy may be particularly important given the highest rate of anastomotic leakage in the curative treatment of esophageal cancer [40]. Moreover, there can be particular translation significance for BPC 157 therapy efficacy. Esophagogastric anastomosis in rats goes with a severe downhill course lethal within not more than 5 days period. This was fully reversed by BPC 157 therapy [40].

Regularly, without therapy, the rats exhibited necrosis and abundant mostly polymorphonuclear infiltration along the anastomosis line, and the inflammation extended to the adipose tissue. The superficial epithelium contained a large necrotic area. There was a broad band of necrotic subcutaneous tissue and muscle. Furthermore, there was advanced esophagitis (i.e., confluent hemorrhagic and yellowish lesions grossly, and ulcerations with pronounced subepithelial and muscular edema, mononuclear infiltration, thinner epithelium, and superficial corneal layers, microscopically) and hemorrhagic lesions in the stomach, some presenting with extensive necrosis to all parts of the mucosa, along with completely failed pyloric sphincters [40]. The dangerous course of the disease was fully counteracted by all BPC 157 regimens from the very beginning. The water volume before anastomosis leakage was more than two times that in the controls. The initial organization of the exudate and the wound being completely closed with granulation tissue could substantiate that the anastomoses were strengthened. Furthermore, as evidence of the advanced esophagogastric healing and the successful re-establishing of the esophagogastric integrity, gastric lesions and esophagitis lesions were attenuated. The failed pyloric sphincter pressure was markedly increased toward normal values. The pressure in the esophagus at the site of the anastomosis appeared similarly to regained sphincter function. Lethal outcomes were completely eliminated. Weight loss was attenuated [40]. Additionally, BPC 157 treatment in combination with L-NAME nullified any effect of L-NAME that otherwise severely aggravated the regular course. The similar effective BPC 157 therapy regimens (/kg) (i.e., intraperitoneally once daily, BPC 157 10 μg, 10 ng, or BPC 157 10 μg, 10 ng in drinking water) support each other through their effect and easy applicability. As a particular highlight, note that, immediately upon anastomosis creation and saline bath application, and at least within the following15 min, the rat gastric surface is without blood vessels. On the other hand, as a sign of the onset of rapid recovery, along with the previously mentioned particular vascular recovering effect that would counteract the imminent perilous course [19,20,21,22,23,24,25,26,27,28,29,30,31,32,33], blood vessels remained present at the gastric surface after anastomosis creation with the BPC 157 bath [40].

To emphasize the significance of the point of the anastomosis special healing in the upper part of the gastrointestinal tract [40], evidences of the healing of various anastomoses in the lower part of the gastrointestinal tract were combined [41,42,43,44,45]. Moreover, the evidence of the colon–colon anastomosis healing and cysteamine colitis by BPC 157 therapy [44,45,46], in particular, follows the clinical evidence of the accomplished ulcerative colitis in clinical phase II [1,2,3,4,5,6,7,8,9,10,11].

## 4. Anastomoses in the Lower Part of the Gastrointestinal Tract

### 4.1. Colocolonic Anastomosis with Cysteamine Colitis

**Figure 1:**
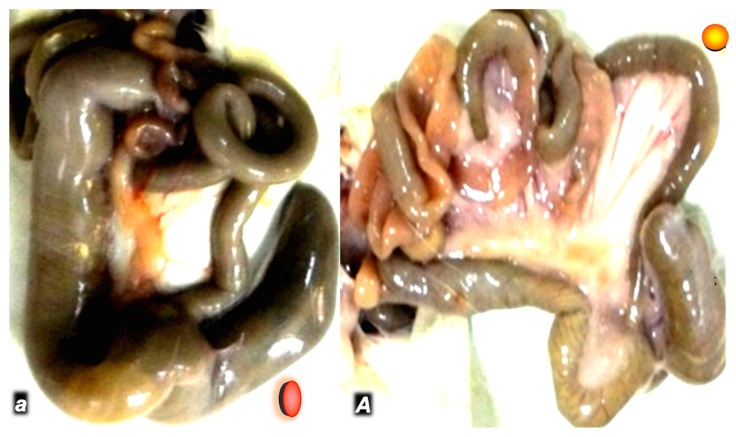
Day 7 post operation in rats that received cysteamine 400 mg/kg enema and colon–colon termino-terminal anastomosis (controls *small italic letters*, red circle; BPC 157 *capital italic letters*, yellow circle) (**a**,**A**). Subileus/ileus in controls (**a**), and intestine presentation close to normal in BPC 157 rats (10 µg/kg/day in drinking water) (**A**).

In a rat study intended to show that the same regimen of BPC 157 can successfully counteract severe disturbances in the gastrointestinal tract and multiple sclerosis-like disturbances, peripherally and centrally, BPC 157 therapy reveals the high potential to heal colon–colon anastomosis along with cysteamine colitis [44] (Figure 1). Therefore, from the viewpoint of the anastomosis healing, this means a therapy action as a whole, the potential to counteract the consequence of the anastomosis for cysteamine colitis, and vice versa, convalescing the increased healing failure of anastomosis healing due to simultaneous presentation of severe colitis [44]. 

In general, grossly, clinically, microscopically, and, in particular, biomechanically, the controls could not heal cysteamine colitis and colon–colon anastomosis, and poor healing occurred [44]. Only a tiny volume without anastomosis leaking could be sustained. Likewise, only a minute quantity of the applied water could sustain their adjacent colon relatively far from anastomosis but with colitis. Contrarily, i.e., less necrosis, increased epithelization, new strands of smooth muscle, more granulation, and a smaller number of inflammatory cells characterized a strong benefit of BPC 157 therapy. Accordingly, a markedly higher intestine strength before leaking was observed (two or three times higher than in controls). This could be illustrated from the seventh post operative day, where they presented values comparable to those of healthy animals. Likewise, BPC 157 therapy evidence of general improvements (i.e., markedly improved post operative course, formed stool, no passage obstruction, markedly fewer adhesions, and maintained rat weight) contrasts with the controls presenting poor conditions with huge adhesions, prominent passage impairment, non-formed stool, and weight loss.

### 4.2. Colocolonic Anastomosis with Occlusion of the Inferior Mesenteric Artery

**Figure 2:**
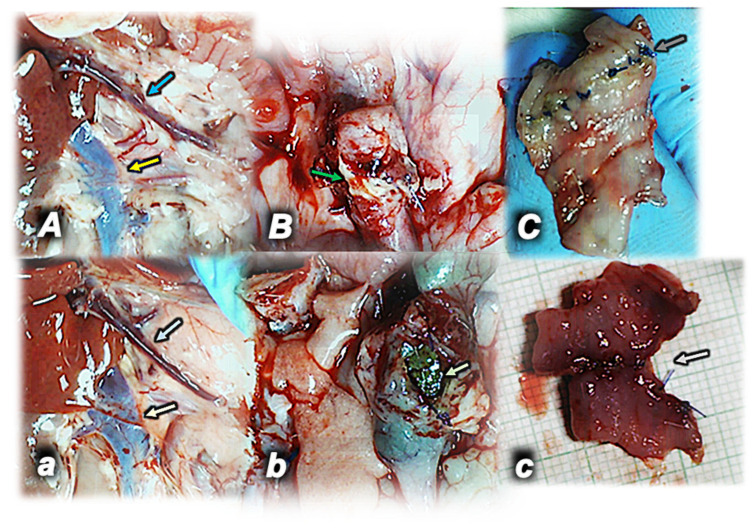
Rats with occlusion of the inferior mesenteric artery and colon–colon anastomosis (**A**,**a**,**B**,**b**,**C**,**c**) in control rats (lower, light color arrows) (*small italic letters*) and in rats that received BPC 157 in drinking water (upper) (*capital italic letters*). Superior mesenteric vein (blue arrows) and inferior caval vein (yellow arrows) congested (control, lower) (**a**) or close to the normal presentation (BPC 157, upper) day 3 post operation (left) (**A**). Anastomosis dehiscence open (control, lower) (**b**) or sealed with adhesion (BPC 157, upper) (**B**) (green arrows) (middle) day 5 post operation. Anastomosis presentation, poor (control, lower) (**c**) and fully healed (BPC 157, upper) (**C**) upon sacrifice day 7 post operation (gray arrows) (right).

A similar failed anastomosis presentation appeared in rats with inferior mesenteric artery occlusion, and they sustained only a very small amount of the water unless BPC 157 therapy was provided [46]. On the other hand, a strong benefit occurred via BPC 157 therapy: these rats being biomechanically assessed demonstrated markedly higher intestine strength before leaking, less necrosis, increased epithelization, new strands of smooth muscle being formed, more granulation, and a smaller number of inflammatory cells. Furthermore, as an additional point, a particular remark was on the congested superior mesenteric vein and inferior caval vein that likely appeared as part of the occlusion/occlusion-like syndrome, which regularly would appear with major injury development (i.e., colon-colon anastomosis along with occlusion of the major artery, i.e., inferior mesenteric artery). This was also counteracted by BPC 157 therapy application, evidencing a general healing effect [46] (Figure 2).

### 4.3. Jejunoileal Anastomosis and Short Bowel

With jejunoileal anastomosis, BPC 157 exhibited the increased breaking strength of anastomosis, and they could sustain a considerably higher volume of leak induction (more than a twofold increase) [41,42]. However, what is more important is the therapy of the short bowel in rats.

The first long-term rat study reports in a particular way by BPC 157 therapy the recovery of the short bowel syndrome in rats and the recovery of rat exhaustion following massive small bowel resection and subtotal jejunoileoectomy [41]. Evidence was collected that indicated that BPC 157 administered intraperitoneally or per-orally (in drinking water) generally improved intestinal adaptation as a result of its particular adaptive effects, i.e., on villus height, crypt depth, and muscle thickness (and inner (circular) and/or outer (longitudinal) muscular layer). In this, a particular point is the habitual adaptation that seems to be successfully collaborated by BPC 157 across the whole 4 weeks [41]. This effect is increasingly and specifically present, point-by-point, for each part of the intestinal wall. Illustrative is the period of extensive adaptation during the first week as well as the later period. During the first week, with more than a fourfold increase in muscle thickness, the inner (circular) muscular layer was further increased by therapy (while the outer (longitudinal) muscular layer was not affected by therapy). Likewise, twofold increase of villus height and crypt depth was additionally increased by therapy. In the later period characterized by preserved villus height and crypt depth and outer (longitudinal) muscular layer thickness, collapsed inner (circular) muscular layer thickness, villus height, and crypt depth were further increased, collapsed inner (circular) muscular layer thickness reversed toward further increased thickness while outer (longitudinal) muscular layer thickness remained unaffected. Consequently, it could be the improved adaptation where each of the increases in villus height, crypt depth, and mucosal muscle thickness (most prominently in inner (circular) muscular layer thickness) was specifically targeted. Therefore, a new improved equilibrium in the remaining intestine layers is observed, along with the observed constant weight gain [41]. Evidently, such particular adaptation is well achieved by parenteral or per oral BPC 157 therapy (the critical inner (circular) muscular layer fully recovered). This means that intestine function (i.e., the same jejunum/ileum diameters (and thereby, no passage disturbances)) was successfully recovered as a whole, given that the BPC 157-treated short bowel rats would finally reach the weight of the normal rats [41].

The next short-term rat study after subtotal jejunoileoectomy verifies this particular use of BPC 157 therapy to recover short bowel syndrome and to recover exhaustion following massive small bowel resection quickly occurring during the first 24 h [42]. Besides the counteraction of poor anastomosis healing and counteraction of the failed intestine adaptation, there was also a counteraction of the concomitant lesions in the gastrointestinal tract, liver, and brain [42]. Moreover, there was also a counteraction of the worsening that appeared with the diclofenac application, as well as a counteraction of the additional worsening that appeared with the application of NO-blocker, L-NAME [42]. Of note, it seems that BPC 157 therapy collaborates even more with the habitual adaptation, given the greatly increased villus height and crypt depth, outer (longitudinal) muscular layer thickness, and inner (circular) muscular layer thickness that would be additionally increased by BPC 157 therapy. Thus, this emphasizes a highly orchestrated effect on the intestinal wall as a whole, specifically and timely related to the healing course, which can result in rats with short bowels to surplus adaptation so that intestine function restoration can be close to normal.

### 4.4. Ileoileal Anastomosis

**Figure 3:**
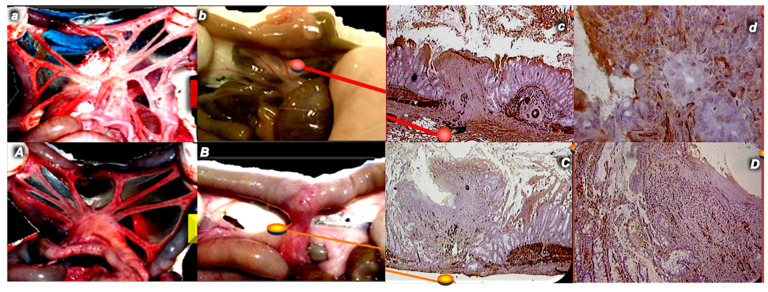
Rats with ileoileal anastomosis (**A**,**a**,**B**,**b**,**C**,**c**,**D**,**d**) in control rats (upper, *small italic letters*, red indices) and in rats that received BPC 157 (per-orally or intraperitoneally) (lower, *capital italic letters*, yellow indices). Vessels presenting as empty distal vessels due to anastomosis (control, upper, (**a**)) or close to the normal presentation, proximal and distal from anastomosis (BPC 157, lower, (**A**)) day 1 post operation (left). Day 14 post operation. Abundant adhesions in controls (lower, (**b**)) or less adhesions in BPC 157 rats (lower, (**B**)) (middle, left). In controls, muscularis mucosa and propria abrupt ending (×2 (**c**), ×25 (**d**), control, upper) was observed; in BPC 157 rats, strands of newly formed muscle (×2 (**C**), ×6.5 (**D**), BPC 157, lower) (right) were observed.

Regularly, the formation of ileoileal anastomosis in rats results in the progression of anastomosis adhesion formation involving many neighboring small intestine loops, the stomach and liver being “packed”, and intestinal passage obstruction [43]. As a cause–consequence course, vessels become empty, and the failed filling of vessels with blood tends to deteriorate. Only small volumes and small pressures that anastomosis could initially sustain consistently remain (i.e., during the first 5 days, they demonstrate no increase) far below healthy values. Consistently, strong edema, necrosis, increases in the number of granulocytes but a poor formation of granulation tissue, reticulin, and collagen, and inadequate epithelization characterize poor healing in the control rats with ileoileal anastomosis [43]. Contrarily, all of the parameters of anastomotic wound healing were significantly improved in BPC 157 rats. Indicatively, edema and the number of granulocytes markedly decreased, and subsequently, (from day 4 or 5) necrosis was attenuated, while granulation tissue, reticulin, and collagen formation substantially increased. The healthy values were already observed by the end of the first week; anastomosis without leakage demonstrated a markedly continuous increase in volume and pressure values. It was immediately effective (in µg-ng range for all parameters, pg-dose attenuated adhesion formation) [43]. Accordingly, with the indicated cause–consequence course, the adhesion formation remained attenuated, mild intestinal passage obstruction was only temporarily observed, and rats presented blood vessels filled with blood (while the empty vessels characterized the controls). Furthermore, there was more new vessel formation in the granulation tissue filling the defect as a part of the promoted angiogenesis. As a particular additional healing point in pentadecapeptide BPC 157 rats, (desmin immunohistochemistry) strands of newly formed muscle in rats with ileoileal anastomosis appeared. Contrarily, an abrupt ending of the muscular layers (both muscularis mucosa and propria) characterized the poor presentation of control rats [43] (Figure 3).

## 5. Other Anastomoses Healing

### 5.1. Sciatic Nerve Anastomosis

Given the suggested innate wound healing capability [2], the focus was on BPC 157 therapy in the healing of rat transected sciatic nerves [47]. In general, BPC 157 rats exhibited faster axonal regeneration histomorphometrically, electrophysiologically (increased motor action potentials), functionally (improved SFI), and with autotomy absent [47]. The presentation of neural fascicles, homogeneous regeneration pattern, density and size of regenerative fibers, existence of epineural and perineural regeneration, uniform target orientation of regenerative fibers, and higher proportion of neural vs. connective tissue were all improved. Furthermore, all fascicles in each nerve showed an increased diameter of myelinated fibers, thickness of the myelin sheet, number of myelinated fibers per area and myelinated fibers as a percentage of the nerve transected area, and blood vessel presentation. Moreover, these effects were obtained using different injury settings, BPC 157 therapy regimens (10 µg, 10 ng/kg), and ways of application (intraperitoneally/intragastrically/locally) with considerable improvement either shortly after injury (BPC 157 given intraperitoneally/intragastrically/locally, at the site of anastomosis) or after non-anastomosed nerve tubing (7 mm nerve segment resected, BPC 157 given directly into the tube). There, as emphasized, the full substantiation of the evidence that BPC 157 markedly improved rat sciatic nerve healing [47] was a very consistent improvement clinically (autotomy), microscopically/morphometrically, and functionally (EMG, one or two months post injury, walking recovery (sciatic functional index (SFI)) at weekly intervals.

### 5.2. Abdominal Aorta Anastomosis

The abdominal aorta anastomosis study [48] applied the long-standing background of the cytoprotection concept, emphasized in the stomach cytoprotection theory [13,14], holding endothelium protection as an innate ability of the cytoprotective agents’ activity. The additional background was that BPC 157 is a pentadecapeptide, being native and stable in human gastric juice for more than 24 h and acting as a cytoprotection mediator [1,2,3,4,5,6,7,8,9,10,11]. Thus, it was likely that BPC 157 therapy can manage the abdominal aorta anastomosis (aortic termino-terminal anastomosis in rats with a formed cloth obstructing more than a third of aortic lumen, severely impaired walking ability, painful screaming, and weak muscle strength) and can counteract the thrombus formation at the site of anastomosis [48]. The therapy was given either to prevent or attenuate thrombus formation (i.e., immediately after anastomosis creation) or to reverse the already formed thrombus at the site of anastomosis (at 24 h after surgery). The recovery of the function of lower extremities was used as the assessment point. After 24 h following aortic termino-terminal anastomosis, we showed that BPC 157 (10 µg/kg) may also decrease the formation of cloth after aortic termino-terminal anastomosis and preserve walking ability and muscle strength when given as a bath immediately after aortic anastomosis creation. Thereby, the effect of BPC 157 (10 µg/kg) was additionally studied at 24 h following aortic termino-terminal anastomosis. Given at that point, intraperitoneally, within 3 min post application interval, the pentadecapeptide BPC 157 rapidly recovered the function of lower limbs and muscle strength, while no cloth could be seen in those rats at the anastomosis site [48].

Note that the point of the direct effect on endothelium to counteract thrombosis [48] was further strengthened. There is evidence that BPC 157 therapy successfully counteracted prolonged bleeding induced by amputation, perforation, and/or anticoagulant and antiplatelet drug application without affecting coagulation pathways. Likewise, there is a direct effect on thrombocyte function maintenance [16,17,18,31]. As definitive proof, in accordance with the wound healing effect mentioned before, there is a counteraction of vascular and multiorgan failure occlusion/occlusion-like syndromes [19,20,21,22,23,24,25,26,27,28,29,30,31,32,33]. Therefore, by activating collateral rescuing pathways, BPC 157 therapy can re-establish reorganized blood flow; BPC 157 therapy counteracted both hemorrhages (i.e., in the brain and lung) and thrombosis in arteries and veins peripherally and centrally, all as part of the counteraction of advanced Virchow triad circumstances [19,20,21,22,23,24,25,26,27,28,29,30,31,32,33].

As such, BPC 157 therapy in gastrointestinal lesions and anastomosis healing providing regularly less adhesion formation [40,41,42,43,44,45,46], with a particular vascular effect, can be suited for the realization of peritoneal defect healing with minimal or no adhesion formation by counteracting adhesion formation and reversing existing adhesions [49]. Specifically, BPC 157 was shown to interfere when two damaged peritoneal surfaces come into contact with each other and reverse the healing that would result in fusion to form a connection, e.g., an adhesion. Given the restoration of normal tissue structure and function obtained along with BPC 157 therapy, this can be achieved by modulating the temporary role of fibrin so that healing without adhesions can occur [49].

In summary, this review of BPC 157 therapy provides evidence for intestinal anastomosis healing [40,41,42,43,44,45,46] and other anastomoses healing (sciatic nerve, abdominal aorta) [47,48] both in general and in particular. Thus, there are limitations (i.e., translation from animal data) and strengths of this review. It would likely not completely resolve the full extent of the still-existing lack of definitive knowledge about the basics of the process of anastomotic wound healing in the gastrointestinal tract [12]. On the other hand, by introducing many distinctive anastomoses healing by BPC 157 therapy, this review practically deals with the concept of the resection and reconnection of particular parts of the gastrointestinal tract [40,41,42,43,44,45,46] as one of the cornerstones of visceral surgery [12]. It may be that the healing of quite distinctive anastomoses by itself speaks for applied BPC 157 therapy, in particular, as a way in which the therapy of anastomoses can be successfully approached and carried out. Esophagogastric, colocolonic, jejunoileal, and ileoileal anastomoses were implicated, along with concomitant disturbances, such as esophagitis, sphincter dysfunction, failed intestinal adaptation, colitis, short bowel syndrome, major vessel occlusion, and NO-system and prostaglandins-system dysfunction, which were accordingly counteracted as well, and, finally, findings concerning the healing of other anastomoses (i.e., nerve and vessel) [40,41,42,43,44,45,46,47,48]. Moreover, the healing of the fistulas, both external and internal, colocutaneous, gastrocutaneous, esophagocutaneous, duodenocutaneous, vesicovaginal, colovesical, and rectovaginal in rats, perceived as anastomoses made between two different tissues that are normally not connected, may be also indicative [3]. This may be a particular reconnection of the parts of the gastrointestinal tract to re-establish adequate integrity depending on the tissue involved, given that both various intestinal anastomoses and various fistulas (intestinal and skin were accordingly healed simultaneously so that fistulas disappeared) were all healed [3,40,41,42,43,44,45,46,47,48]. Furthermore, to ascertain undisturbed anastomoses healing, BPC 157 therapy has a strong antitumor potential (i.e., counteracting the VGEF tumor promoting effect) [50] and can counteract tumor-induced cachexia [51]. Together, these findings provide a tightly interconnected network of evidence that can hardly be disputed.

## 6. Conclusions

BPC 157 therapy applied to intestinal anastomoses can be quite effective and safe. This can be observed in the resolved intestinal anastomoses (both in the upper and the lower gastrointestinal tract (i.e., esophagogastric, colocolonic, jejunoileal, and ileoileal anastomoses)), the resolved healing of other anastomoses (i.e., nerve and vessel), and the resolved concomitant disturbances (i.e., esophagitis, sphincter dysfunction, failed intestinal adaptation, colitis, short bowel syndrome, major vessel occlusion, dysfunction of the NO-system and prostaglandins-system) [40,41,42,43,44,45,46,47,48]. Given the complexity of intestinal and other anastomoses healing, the particular mechanism(s) behind should be further revealed, as BPC 157 communicates with different molecular pathways [1,36,37,51,52,53,54,55,56,57,58,59], particularly with the NO-system. However, for practical purposes, despite the necessity for further research, it should be noted that the tensile strength of the anastomosis should be a direct reflection of the successful repair process [3,40,41,42,43,44,45,47,48], regardless of mechanism(s). The same findings were also noted in skin, muscle, tendon, and ligament healing [11]. Finally, it can be safe to conclude that BPC 157 therapy presents a practical resolution of the major problems of anastomosis healing, the healing of different tissues that otherwise tend to heal slowly, and impaired medication accessibility.

Finally, BPC 157/anastomosis healing conceptually related to a regulatory physiologic role in bodily functions [60] due to the large presence in human fetuses and adult tissues (in situ hybridization and immunostaining) [60] and based on the similar beneficial effects in other species (i.e., birds [61] and insects [62,63,64]). Very recently, within the concept of cytoprotection, the particular aspects of the stable gastric pentadecapeptide BPC 157 pleiotropic beneficial activity [1,2,3,4,5,6,7,8,9,10,11] were observed to have a relationship with neurotransmitters’ activity [65]. Also, within the cytoprotection concept—as born by Robert (epithelium) [13] and Szabo (endothelium) [14] (i.e., performing innate stomach integrity maintenance that should regularly be translated to other organ healing, as well as through cytoprotective agents’ application [13,14,15])—BPC 157, being native and stable in human gastric juice, as a hormone-like activity, can be released into the circulation and sent to distant organs by complex biological processes to regulate physiology and behavior [65]. The final advantage is also a very safe BPC 157 profile (i.e., no adverse effects in clinical trials (ulcerative colitis, phase II), and in toxicological studies, LD1 could be not achieved) [1,2,3,4,5,6,7,8,9,10,11], a point recently confirmed in a large study conducted by Xu and collaborators [66]. Together, these BPC 157/anastomosis healing relations [3,40,41,42,43,44,45,46,47,48] might be suggestive of further applications of BPC 157 therapy.
